# A20 in Kidney Transplantation and Autoimmunity

**DOI:** 10.3390/ijms25126628

**Published:** 2024-06-16

**Authors:** Andreas Kommer, Myriam Meineck, Paul Classen, Julia Weinmann-Menke

**Affiliations:** Department of Nephrology, I. Department of Medicine, University Medical Center Mainz, Johannes Gutenberg University, D 55131 Mainz, Germany; myriam.meineck@unimedizin-mainz.de (M.M.); paul.classen@unimedizin-mainz.de (P.C.)

**Keywords:** A20, TNFAIP3, kidney transplantation, autoimmunity

## Abstract

A20, the central inhibitor of NFκB, has multiple anti-inflammatory properties, making it an interesting target in kidney autoimmune disease and transplant biology. It has been shown to be able to inhibit inflammatory functions in macrophages, dendritic cells, T cells, and B cells in various ways, leading to less tissue damage and better graft outcomes. In this review, we will discuss the current literature regarding A20 in kidney transplantation and autoimmunity. Future investigations on animal models and in existing immunosuppressive therapies are needed to establish A20 as a therapeutic target in kidney transplantation and autoimmunity. Cell-based therapies, modified viruses or RNA-based therapies could provide a way for A20 to be utilized as a promising mediator of inflammation and tissue damage.

## 1. Introduction

Kidney disease is a common condition affecting around 10% of the population worldwide and it is projected to be the fifth most common cause of life years lost by 2040 [1,2]. Although the most frequent cause is diabetes mellitus, glomerulonephritis, mostly due to autoimmune disease, is usually among its top causes [3]. If kidney disease progresses to end-stage kidney disease (ESKD), organ replacement therapy is indicated. The best option regarding patient independence and clinical outcomes is kidney transplantation [4].

A better understanding of autoimmune disease and its causes could prevent kidney disease and organ failure by leading to better targets for therapy. If a kidney transplantation is necessary, delayed graft function (DGF) is an important predictor of long-term transplant outcome and there are currently no therapeutic options to influence its occurrence [5]. Moreover, acute and chronic allograft rejection, whether they are T cell or antibody mediated, are the most frequent factors limiting graft function [6]. Due to its anti-inflammatory properties for different levels of the immune system, the cytoplasmic enzyme A20 has become a promising target for all the aforementioned problems in kidney disease. This review aims to provide an overview of the latest findings on the role of A20 in kidney transplantation and autoimmunity.

## 2. Review

### 2.1. Molecular Interactions of A20

A20 is a cytoplasmic ubiquitin-editing enzyme encoded by the tumor necrosis factor-α (TNF)-induced protein 3 (TNFAIP3) gene, which is situated on chromosome 6q23 and counts 790 amino acid residues [7,8]. It was first described in 1990 in endothelial cells after stimulation with TNF, interleukin-1 (IL-1) and lipopolysaccharide (LPS) [9]. One of its central functions is the inhibition of nuclear factor kappa B (NFκB) signaling [10]. Independent of NFκB, A20 can suppress apoptosis and, additionally, has regulating functions in the NOD-like-receptor protein 3 inflammasome (NLRP3), the wingless pathway (wnt) and in interferon signaling (IRF) [11,12,13]. All of these functions make A20 a key anti-inflammatory molecule.

#### 2.1.1. Structure of A20

**Figure 1:**
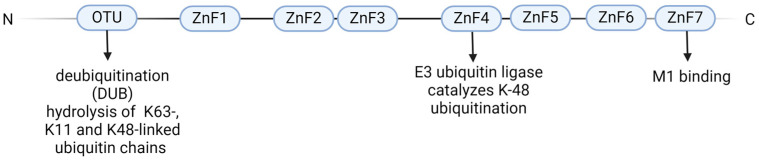
Structure of A20.

A20 has eight domains, many of which are involved in ubiquitin-editing functions (Figure 1) [11,14]. The so-called OTU (ovarian tumor) domain is located at the N-terminus and mediates deubiquitination (DUB) by hydrolysis particularly of the K63-linked but also of the K11- and K48-linked ubiquitin chains [14,15]. C-terminally, there are seven zinc finger (ZnF) domains, of which ZnF4 and ZnF7 are the most important. ZnF4 has an E3 ubiquitin-ligase function and catalyzes K-48 ubiquitination, which promotes the degradation of the target molecules [16]. For example, A20 can remove K63-linked ubiquitin catalyzed by the OTU domain from the receptor interacting protein 1 (RIP1), a key mediator of the TNF receptor 1 (TNFR1) signaling pathway, thereby inactivating it and, in a second step, cause K48 ubiquitination and thus proteasomal degradation [17]. In this way, TNF-induced activation of NFκB is inhibited. By binding to linear ubiquitin chains, ZnF7 contributes to its recruitment to receptor complexes, which stabilizes them and inhibits downstream inflammation [18]. The key mechanism on which A20 acts is a ubiquitin-editing enzyme, via its zinc finger domains.

#### 2.1.2. A20 as Central Inhibitor of NFκB

NFκB is a transcription factor activating around 400 genes, which induce the production of cytokines, chemokines, acute-phase proteins and cell-cycle proteins [19,20]. It is a central molecule for the activation of the innate and adaptive immune system to protect against infections. It triggers the formation of pro-inflammatory mediators such as TNF, IL-1b and interleukin-6 (IL-6) and represses the formation of anti-inflammatory mediators like interleukin-4 (IL-4). Known NFκB inducers are reactive oxygen species (ROS), TNF, interleukin-1, bacterial lipopolysaccharides (LPS) and endothelin-1 [21].

Improper activation of NFκB, in turn, can cause chronic inflammation leading to autoimmune disease [22]. Disturbed regulation of NF-κB by A20 is also involved in oncogenesis. Studies have shown that A20 is able to inhibit tumor growth and that A20 defects are common in various types of cancer such as lymphoma, melanoma and gastrointestinal tumors [23,24]. Impaired control of the NFκB signaling pathway by A20 can also lead to increased susceptibility to infections and immune incompetence [25]. NFκB is activated via various receptor stimuli including the TNF-receptor complex, the Toll-like receptor/IL-1β (TLR) receptor, the Interleukin 17 (IL-17) receptor, and many others [12,26]. After activation, NFκB induces transcription of TNFAIP3 and its activation via phosphorylation by the inhibitor of κB (IκB) kinase (IKK) [27,28,29]. A20 then, in turn, inhibits NFκB signaling by inhibiting the nuclear factor κB essential modulator (NEMO) via IKK impairment and in numerous other ways, making A20 a key player in the negative feedback loop of NFκB signaling [26,30].

#### 2.1.3. A20 in Different Receptor Cascades

A20 has been shown to interact with several signaling cascades of different receptors. As mentioned above, in TNF-receptor stimulation, A20 inhibits TNF receptor-mediated NFkB activation by interacting with RIP1. Both the Toll-like receptors (TLRs) and IL-1R mediate their signals through myeloid differentiation primary response 88 (MyD88), IL-1 receptor associated kinase (IRAK) and tumor necrosis factor receptor associated factor 6 (TRAF6). A20 causes the removal of K63-linked polyubiquitin from TRAF6, thereby inhibiting TLR/IL-1R signaling [7]. In addition, A20 can inhibit the E2 enzymes UBCH5 and UBC12 and thereby the E3 ligase activity dependent on them [7]. Based on this mechanism, both TNFR1-dependent and MyD88/IRAK/TRAF6-dependent NFkB activation is reduced. Moreover, A20 can restrict mitogen-activated protein kinases (MAPKs) by hindering phosphorylation of c-Jun N-terminal kinases (JNKs) after stimulation of TLR or IL-17R, and therefore lower the production of IL-17 in Th17 cells [30,31]. A20 also affects interferon receptor signaling. The absence of A20 increases STAT1-dependent inflammation and A20 represses STAT1 transcription through IFNβ-dependent and NFκB-independent mechanisms [32,33]. Overall, A20 acts as a negative feedback in all of the mentioned receptor cascades, making it an interesting target for immune modulation.

#### 2.1.4. A20 Protects Cells from Death

A20 can protect cells from cell death by apoptosis and necroptosis, in different ways [14]. A key mechanism to protect against programmed cell death is the influence of TNF-induced apoptosis. In order for TNF signaling to lead to cell death, various molecular brakes must be inactivated [34]. When RIP1 dissociates from the TNF receptor and becomes part of the so-called complex II, it can induce apoptosis or necroptosis. Four checkpoints have been described [34]. A20 can prevent Complex II formation by preventing cylindromatosis (CYLD)-mediated deubiquitinylation of Complex I [11]. In addition, A20 can inhibit death-inducing signaling complex (DISC) formation due to activation of the TNF-related apoptosis-inducing ligand (TRAIL) receptor by deubiquitylating caspase-8 [11,35]. By ubiquitinylating RIP1, it can also enable its recruitment to DISC and thus impair the activation of caspase-8 [36,37]. Nevertheless, proapoptotic influences of A20 have also been described [38,39]. The inhibitory function of A20 for TNF-induced apoptosis makes it an interesting target in inflammation.

#### 2.1.5. A20 Effect on Different Cell Populations

A20 has shown to influence several cell types of the innate and adaptive immune system. In macrophages, A20 inhibits Interleukin-33-induced signal transducer and activator of transcription 1 (STAT1) signaling, leading to reduced interferon-γ (IFN-γ) production and increased anti-inflammatory IL-4-mediated STAT6 signaling [40]. In addition, it suppresses NLRP3-mediated M1 polarization of macrophages, shifting the balance more towards the anti-inflammatory M2 subtype [41,42]. In a murine model of arthritis, A20 prevented disease by inhibiting macrophage necroptosis, likely through the mechanism mentioned above [43].

In CD4^+^ T cells A20 promotes autophagy after TCR stimulation and binds to the mammalian target of rapamycin (MTOR) inhibiting its activity [44]. In CD8^+^ T cells, A20 negatively controls the liberation of IFN-γ and IL-2 [45]. It also restricts the expansion of pro-inflammatory Th17 cells [46]. Overall, this shifts the balance from inflammatory T cells to regulatory FoxP3^+^ T cells [47].

In B cells, A20-deficiency leads to proliferation, development of autoantibodies and increased expression of IL-6 [48,49]. This is likely due to resistance to Fas-mediated cell-death caused by increased NFκB-induced overexpression of anti-apoptotic proteins [50]. This has led to A20 being investigated in antibody-mediated diseases such as systemic lupus erythematosus [49,50].

**Figure 2:**
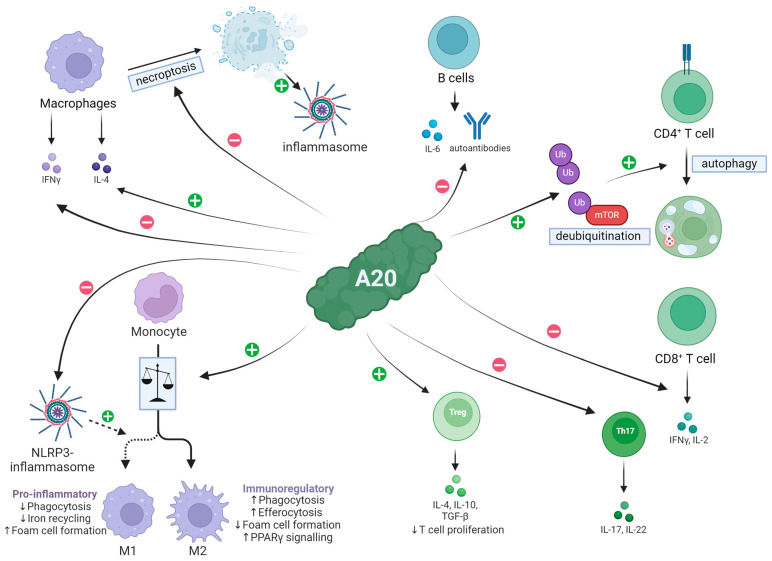
A20 effect on different cell populations. A20 causes a reduced IFNy release in macrophages, while the anti-inflammatory IL-4 production is increased. Furthermore, it inhibits macrophage necroptosis, which promotes arthritis in mice via inflammasome activation. In addition, A20 promotes a shift in the polarization of macrophages towards the anti-inflammatory M2 subtype. The suppression of NLRP3-mediated M1 polarization also supports this. A20 promotes the expression of regulatory T cells and thus simultaneously suppresses the expression of pro-inflammatory T cells such as Th17 cells. Furthermore, the expression of proinflammatory cytokines by T cells is inhibited. A20 promotes autophagy of CD4^+^ T cells by binding to it and probably contributes to its deubiquitinylation. With regard to B cells, A20 causes a lower expression of IL-6 and autoantibodies. IL = Interleukin, IFN = Interferon, NLRP3 = NOD-, LRR- and pyrin domain-containing protein 3, PPAR = Peroxisome proliferator-activated receptors, TGF-β = Transforming growth factor β, mTOR = mammalian target of rapamycin.

Because of its anti-inflammatory effect on macrophages, T cells and B cells (Table 1 and Figure 2), A20 has long been a target of research into transplantation and autoimmune kidney disease.

#### 2.1.6. A20 in Autoimmunity

Since its discovery, A20 impairment has been linked to numerous autoimmune diseases. Mice with a complete A20 knockout are unable to survive, and die shortly after birth as a result of severe autoinflammation affecting various organs [51]. Reduced A20 expression is connected to many autoimmune diseases, such as systemic lupus erythematosus (SLE), inflammatory bowel disease (IBD), type 1 diabetes, and rheumatoid arthritis, as well as ankylosing spondylitis and Sjörgen syndrome [52,53]. Additionally, the most commonly used anti-inflammatory drugs (glucocorticoids) upregulate the expression of A20, making it an interesting focus of research [54]. Binding of the glucocorticoid receptor to an intronic enhancer of the TNFAIP3-gene locus can maintain prolonged A20 expression even after the reduction of NFκB [54,55]. It has been discovered that heterozygous loss-of-function mutation in TNFAIP3 causes an auto inflammatory disease called “A20 haploinsufficiency” [56]. It is a disease similar to Behçet disease, causing oral, genital and/or gastrointestinal ulcers. Its onset is often very early in life and it can lead to severe end organ damage and even death [57]. There are various mouse models that can be used to study A20 in autoimmune diseases (Table 2).

#### 2.1.7. A20 in Systemic Lupus Erythematosus (SLE)

In humans, genome analysis has linked multiple TNFAIP3 polymorphisms to SLE, with some being especially prominent in Lupus nephritis (LN) and hematological manifestations [58,59,60,61,62]. Several single-nucleotide polymorphisms (SNPs) affecting the A20 coding region have been shown to increase the risk of SLE [58,63]. The mutation affecting the A20 DUB domain has been shown to promote the expression of PAD4 [64]. The latter regulates protein citrullination and NET formation, and could thus increase the risk of SLE [64]. Mechanistically, an influence of A20 on B-cell survival could also contribute. Selective deletion of A20 in murine B cells causes enhanced proliferation and excessive production of self-reactive autoantibodies [49]. Mice whose B cells do not express A20 show more glomerular immunoglobulin deposits [50]. A20 limits the survival of activated B cells by NFκB-dependent, reduced expression of antiapoptotic proteins, and can thus reduce autoimmunity [50]. Dendritic cells (DCs) are important in the pathogenesis of SLE due to their antigen presentation and cytokine expression. A20-deficient DCs promote autoimmunity by lowering their activation threshold, persisting longer due to increased resistance to apoptosis and causing plasma-cell conversion in the absence of T cells [65]. In peripheral blood mononuclear cells (PBMCs) of SLE patients, A20 expression is significantly reduced compared to healthy controls and is correlated inversely with disease activity [66]. Stimulation of the PBMCs of SLE patients with TNF resulted in lower expression of A20 when compared with healthy controls [67]. Lupus-prone MRL/lpr mice show reduced A20 expression and this could thus exacerbate lupus nephritis through increased NFκB activation [68]. In murine podocytes, A20 deficiency causes increased cell injury [68].

Due to the fact that mice with A20-deficient B cells show more glomerular immunoglobulin deposits, A20 could be an interesting target for all other antibody-mediated glomerular diseases, such as anti-glomerular basement membrane disease, IgA nephritis, membranous nephropathy, or membranoproliferative glomerulonephritis, in addition to the prototypical antibody-mediated disease SLE.

#### 2.1.8. A20 in Other Autoimmune Diseases

A20-deficient dendritic cells in mice show spontaneous activation and expansion of T cells, causing lymphocytic-dependent colitis, seronegative ankylosing arthritis and enthesitis, similar to human forms of IBD [69].

A meta-analysis with trial sequential analysis (TSA) also showed polymorphisms for rheumatoid arthritis (RA) (especially rs2240926 and rs5029937), which are closely associated with RA [70]. A20 expression is reduced in monocytes from patients with rheumatoid arthritis [71]. Furthermore, there was a correlation between reduced A20 in non-classical monocytes and bone destruction, which is why A20 could attenuate their osteoclastic effect [71]. C57BL/6 mice with A20-deficient myeloid cells develop spontaneous polyarthritis. Increased inflammatory cytokines can be detected in the serum, and macrophages produce more TNF in response to LPS stimulation, in line with an increased NFκB effect. However, the destructive polyarthritis was Toll-like receptor signaling- and IL-6 dependent but TNF independent [72]. Additionally, enhancing A20 expression using an adenovirus in mice with collagen-induced arthritis resulted in reduced tissue destruction [73].

Little is known about the role of A20 in vasculitis, although risk-increasing polymorphisms have been described for both large-vessel and small-vessel vasculitis [74,75]. A20-related SNPs were also identified for systemic sclerosis, which, interestingly, were associated with increased fibrosing alveolitis and pulmonary arterial hypertension, making A20 a possible risk marker for a leading cause of death in systemic sclerosis [76].

In an ovalbumin (OVA)-induced allergic-asthma model in mice, it was shown that overexpression of A20 by adenovirus attenuates the recruitment of airway inflammatory cells and peribronchiolar inflammation and suppresses the production of various cytokines in bronchial secretions [77]. IL-33, known as a key mediator in the pathogenesis of allergic diseases such as asthma or atopic dermatitis, causes increased STAT1 signaling and IFN-γ expression in mice with A20-deficient macrophages, as already mentioned [40].

SNPs have also been described in the context of multiple sclerosis (MS); a significant association of rs10499194, which is located in the intergenic region upstream of TNFAIP3, with MS was found [78]. A20 expression was lower in monocytes and CD4^+^ T cells in therapy-naive MS patients compared to healthy controls, although there was no correlation with disease activity [79]. These data indicate a role for A20 in inflammatory processes of MS.

In inflammatory bowel disease, A20 overexpression by intravenous plasmid vector injection after colitis induction in mice results in reduced colitis activity [80]. Possible mechanisms include downregulation of NFκB and STAT3 activation, as well as reduced IL-17 expression in splenic CD4^+^ T cells [80]. Treatment with a recombinant eukaryotic vector as the nanoparticle causes less colon tissue damage as well as less inflammation through suppression of both NFkB and mucosal mitogen-activated protein kinase (MAPK) [81]. Furthermore, Foxp3 expression and, consequently, the proliferation of regulatory T cells (Treg) are promoted [81]. In patients suffering from Crohn’s Disease, TNFAIP3 mRNA levels in non-inflamed colonic mucosa were significantly decreased compared to healthy controls. In this context, A20 has been suggested to be a potential biomarker [82].

All this taken together underlines the fact that A20 is a promising target in various autoimmune diseases.

#### 2.1.9. A20 in High-Glucose Environments

High-glucose environments cause chronic systemic inflammation comparable to autoimmune diseases, leading to macro- and microvascular disease [83]. High-glucose environments have been shown to cause proteasomal degradation of A20, e.g., via O-glucosamine-N-acetylation of A20, and to subsequently lead to inflammation [28,84]. Overexpression of A20 in the aortic arch of diabetic ApoE-null mice via gene transfer using an adenovirus alleviates accelerated atherosclerosis [84]. Recently it was shown that overexpression of A20 in endothelial cells promotes endothelial nitric oxide synthase (eNOS) transcription and thus counteracts endothelial dysfunction [85].

This is making A20 an interesting target for complications associated with diabetes mellitus, which are likely caused by chronic inflammation, including kidney disease and atherosclerosis.

**Table 2:** A20 animal models for autoimmune disease.

Model	Result
Selective A20-deficient B cells in mice	Enhanced proliferation and excessive production of autoantibodies leading to glomerular deposits, similar to SLE [49,50]
Selective A20-deficient dendritic cells in mice	Activation and expansion of T cells leading to lymphocytic colitis, arthritis and enthesitis [69]
Selective A20-deficient myeloid cells in mice	All mice developed severe arthritis [72]
Enhanced A20 expression in mice with collagen-induced arthritis	Reduced tissue destruction [73]

### 2.2. A20 in Transplant Biology

#### 2.2.1. A20 in Delayed Graft Function

Ischemia and reperfusion injury (IRI) is a well-known risk factor for delayed graft function (DGF) in organ transplantation, and also influences the long-term graft outcome [86,87]. IRI is a mediated injury mainly caused by activating NFκB through TLR and TNF signaling [88,89]. Animal models are used to simulate organ damage and the consecutive inflammatory response in organ transplantation. Upregulating A20 via an adenovirus has been shown to reduce pro-inflammatory activation in endothelial cells, increasing the expression of vascular cell adhesion molecule 1 (VCAM-1), activating NFκB and thereby reducing acute tubular necrosis in rats [90]. Overexpression of A20 by infection of a liposome-wrapped plasmid in rats causes lower retention parameters and less histopathological damage, as well as less apoptosis of proximal tubular epithelial cells (TECs) and inhibition of NFκB activity [91]. In mice, IRI has been shown to cause downregulation of transient receptor potential cation channel-6 (TRPC6), which reduces protective zinc ion influx in TEC, resulting in less A20 expression. This leads to increased activation of the NLRP3 inflammasome and increased IRI [92]. When comparing IRI in mechanically perfused rabbit kidneys with cold-stored rabbit kidneys, higher levels of A20 and lower levels of NFκB and TNF were found in perfused kidneys, leading to fewer inflammatory lesions and less necrosis [93]. Recently, a genetic variant of TNFAIP3 was shown to modulate NFκB signaling and limit IRI in mice by reducing apoptosis, altering the active redox state and increasing the number of regulatory Foxp3^+^-T cells [47]. TECs have been shown to respond to hypoxia by the suppression of A20, leading to a pro inflammatory state [94]. In human kidney biopsies taken 15 min after reperfusion during kidney transplantation, the high expression of TNF, transforming growth factor (TGF)-βCD25, intercellular adhesion molecule-1 (ICAM-1) and IL-10, as well as A20, was associated with delayed graft function likely due to upregulated NFκB signaling [95].

Again, this likely shows A20 as a negative-feedback mechanism compensatory to NFκB signaling. This research highlights the potential of A20 as a protective mediator in IRI.

#### 2.2.2. A20 in Acute and Chronic Rejection

In animal models of solid organ transplantation and human tissue samples of transplanted organs, A20 expression is related to graft outcome (Table 3). Transplant vasculopathy results in chronic graft rejection, while A20 expression of endothelial cells (ECs) and smooth muscle cells (SMCs) correlates with the absence of transplant vasculopathy in rat kidney allografts and long-term function of human kidney allografts [96]. This shows that A20 protects ECs from apoptosis by TNF, Fas and natural killer cells, as it inhibits the proteolytic cleavage of caspase 8 and, furthermore, protects ECs against complement-mediated necrosis. [96]. Adenovirus-induced overexpression of A20 in rats with liver transplantation led to better allograft function, lower transplant fibrosis and longer survival [97]. In rat kidney transplants, a high expression of A20 was associated with lower infiltrates and better vascular integrity, likely caused by lower NFκB-mediated vascular inflammation, which in turn might cause less chronic allograft rejection [98]. A20-haploinsufficient mice receiving aortic transplants show higher infiltration of IFNγ-producing Th1 cells and CD3^+^ T cells, combined with lower numbers of Foxp3^+^ regulatory T cells, leading to more intima hyperplasia [99]. In another model of aortic allograft in mice, using an adenovirus to upregulate A20, lower numbers of Th1/Th17-cell infiltration and higher numbers of regulatory CD25/FoxP3^+^ T cells lead to reduced neointimal formation and arteriosclerosis [100].

Looking for desirable genetic modifications of xenografts, the knockout of α1,3-galactosyltransferase in combination with the increased expression of heme oxygenase-1 and A20 in pigs showed no signs of rejection after four hours of ex vivo perfusion with human blood [101].

During acute allograft rejection, A20 is upregulated in the kidney transplants and can thus prevent kidney damage [102]. This is probably mediated by the negative feedback mechanism on NFκB, as previously described. In intraoperative biopsies, high A20 expression correlated with DGF but not with acute rejection, maybe caused by upregulation of NFκB as a mediator of IRI [103].

A20 could play a protective role in IRI-mediated DGF, for acute but mostly chronic rejection.

In a rat kidney transplant model for chronic allograft rejection, the expression of A20 was significantly higher in the rats receiving mycophenolate mofetil (MMF) as immunosuppressive therapy compared to in those receiving cyclosporine (CsA) [104]. This could be an explanation of how MMF influences transplant rejection and transplant atherosclerosis. In addition, the comparison of sirolimus versus tacrolimus shows that A20 is increasingly expressed in the sirolimus group in a rat model of chronic allograft nephropathy [105]. This highlights the need to evaluate existing immunosuppressive therapies for A20 expression in different tissues and cell populations. 

**Table 3:** A20 animal models in transplantation.

Model	Result
A20 overexpression in rat liver transplantation	Better allograft function, lower transplant fibrosis and longer survival [97]
Rat kidney transplantation	High A20 expression was associated with fewer infiltrates and less vascular inflammation [98]
A20-haploinsufficient aortic-transplant mice	Higher infiltration of CD3^+^ T cells and IFNγ-producing Th1 cells, fewer FoxP3^+^ T cells, more intima hyperplasia [99]
A20 overexpression in aortic-transplant mice	Lower Th1/Th17 infiltration, higher number of FoxP3^+^ T cells, less neointimal formation and arteriosclerosis [100]
Rat kidney transplant with chronic rejection	A20 expression was significantly higher when treated with MMF compared to CsA [104]

## 3. Conclusions

### 3.1. Possible Roles of A20 in Kidney Autoimmunity

A20 seems to be a promising target in kidney autoimmune disease, especially in LN. Its ability to inhibit dendritic cells, which are essential for the activation of autoreactive T cells in LN, could be a potential mechanism for intervening in the early steps of LN pathogenesis [69,106,107]. Furthermore, IL-17-producing Th17 are known to be a key driver of kidney inflammation in LN [106,108]. Since A20 is able to inhibit IL-17 production in Th17 cells [30,31] and inhibit their expansion, upregulating it in kidney tissue could be a viable mechanism to mediate damage [46,47]. Lastly, the nature of A20 deficiency in B cells, resulting in proliferation, hypergammaglobulinemia and autoantibody production, key features of SLE, is worth further studying in SLE [48,49]. These considerations are backed by the genome studies linking A20 polymorphism to SLE disease and the inverse correlation of A20 with SLE disease activity in patients [52,66].

### 3.2. Possible Roles of A20 in Kidney Transplantation

In kidney transplantation, the ability of A20 to reduce pro-inflammatory activation of epithelial cells and reduce acute tubular necrosis in IRI could possibly provide a mechanism to avoid DGF [90,94]. As is the case in autoimmune tubulitis, tubulitis within rejection is also in part caused by IL-17-producing Th17 cells [6]. As described above, the inhibitory function of A20 in IL-17/Th17-induced inflammation could provide a useful target for guided therapies. By limiting graft atherosclerosis and inhibiting macrophage TGF-β secretion [40,98,99], a known driver of transplant fibrosis [109], upregulation of A20 could enhance long-term graft survival. Therefore, A20 is a promising target in kidney transplant biology in IRI and DGF, rejection immunology and long-term transplant fibrosis. Translational studies transferring these in vitro findings into patients are much needed. 

### 3.3. Future Research into A20

Even though all these different functions of A20 are known today, studies on its use as a therapeutic target in humans are lacking. The reasons for this might be the difficulty of upregulating A20 in targeted tissues or cell populations in vivo in humans or its possible role as an oncogene in breast cancer, gastric cancer or melanoma [24]. Nevertheless, interventional trails in animal models of LN and kidney transplantation should be the next step in investigating A20 anti-inflammatory potential.

Overexpression of A20 is an exciting and promising approach as a new therapeutic option for autoimmune disorders. Various approaches are conceivable here.

Modified viruses or in vitro modulated cell populations, similar to chimeric antigen receptor (CAR) t cells, should be used to investigate its ability to modify outcomes. Alternatively modified viruses, similar to vaccines or RNA-based therapies, could be used to upregulate A20 in a desirable fashion. Selective mRNA delivery to different immune cells could represent an interesting approach for various autoimmune diseases. The RNA-based vaccinations against severe acute respiratory syndrome coronavirus 2 have shown that lipid nanoparticles (LNPs) can introduce mRNA into dendritic cells (DCs). In SLE, for example, DCs have been shown to play a key role in pathogenesis [110]. Targeted overexpression of A20 in DCs could be a promising approach here. Similarly, temporary treatment during acute rejection of a kidney transplant could be a potent approach.

Existing biobanks for transplant patients could be used to translate the findings of A20s role in IRI and DGF, rejection immunology and long-term transplant fibrosis into human subjects.

Pharmacologic studies are necessary to find ways to upregulate A20 in different tissues and cells, to explore possibilities in human subjects. First studies on patients under existing immunosuppressive therapy analyzing the effects different agents such as MMF, calcineurin inhibitors or mTOR inhibitors have on the expression of A20 are needed. This could show how existing agents can be used in a more targeted fashion to reduce the use of “unspecific” therapies like glucocorticoids. The ability of different agents to upregulate A20 could be a key to glucocorticoid-sparing therapies. 

Thirty years after its discovery, A20 could be a very promising target in autoimmune disease and transplant biology. 

## Figures and Tables

**Table 1:** A20 effect on different immune-cell populations.

Cell Population	Effect of A20
Macrophages	Reduced IFN-γ production, increased IL-4 secretion [40]; suppresses M1 polarization and necroptosis [41,42,43]
CD4^+^ T cells	Promotion of autophagy and MTOR inhibition [44]
CD8^+^ T cells	Inhibition of IFN-γ and IL-2 secretion [45]
Th17 cells	Restricted proliferation; restraining activity [46]
FoxP3^+^ T cells	Increased influx in tissue [47]
B cells	Decreased proliferation and IL-6 expression; A20 deficiency causes autoantibody production [48,49]
